# Embracing noise to improve cross-batch prediction accuracy

**DOI:** 10.1186/1752-0509-6-S2-S3

**Published:** 2012-12-12

**Authors:** Chuan Hock Koh, Limsoon Wong

**Affiliations:** 1 NUS Graduate School for Integrative Sciences and Engineering, Singapore 117597; 2 School of Computing, National University of Singapore, Singapore 117417

## Abstract

One important application of microarray in clinical settings is for constructing a diagnosis or prognosis model. Batch effects are a well-known obstacle in this type of applications. Recently, a prominent study was published on how batch effects removal techniques could potentially improve microarray prediction performance. However, the results were not very encouraging, as prediction performance did not always improve. In fact, in up to 20% of the cases, prediction accuracy was reduced. Furthermore, it was stated in the paper that the techniques studied require sufficiently large sample sizes in both batches (train and test) to be effective, which is not a realistic situation especially in clinical settings. In this paper, we propose a different approach, which is able to overcome limitations faced by conventional methods. Our approach uses ranking value of microarray data and a bagging ensemble classifier with sequential hypothesis testing to dynamically determine the number of classifiers required in the ensemble. Using similar datasets to those in the original study, we showed that in only one case (*<*2%) is our performance reduced (by more than -0.05 AUC) and, in *>*60% of cases, it is improved (by more than 0.05 AUC). In addition, our approach works even on much smaller training data sets and is independent of the sample size of the test data, making it feasible to be applied on clinical studies.

## Introduction

Noise has a negative connotation in the classical view of biology. Therefore, one often attempts to remove "noise" from data using various statistical methods before any downstream analysis. However, there are two different types of noise in biological data, experimental noise and inherent cell variation. Distinguishing experimental noise from natural fluctuation due to inherent cell variation is a daunting task, and attempts to de-noise data often remove meaningful cell variation as well. Therefore, in this work, we take a different approach of embracing noise instead.

Inherent cell variations could arise from intrinsic and extrinsic sources [1]. Intrinsic noise sources would affect two equivalent and independent gene reporters placed in the same cell differently, whereas extrinsic noise sources would affect two reporters in any given cell equally but affect reporters in another cell differently. Examples of intrinsic noise sources are stochastic events during the process of gene expression, such as transcription regulation, translation regulation and protein degradation. Sources of extrinsic noise include local environmental differences or ongoing genetic mutations. These inherent cell variations have been gaining recognition in their contribution to cell robustness, which enables organisms to survive in the ever-changing environment [1-4].

Experimental noise in gene expression measurement data mainly contains two forms of experimental errors: measurement errors and batch effects. Measurements errors in gene expression microarrays are studied by the MicroArray Quality Control (MAQC) project, a large-scale study led by FDA scientists involving 137 participants from 51 organizations, where they showed that the median coefficient of variation of replicates is between 5% and 15% [5]. The batch effects problem is a non-biological systematic bias that exists in various batches of samples due to experimental handling. If not appropriately handled, incorrect conclusions might be drawn, especially when batch effects are correlated with an outcome of interest [6].

An important application of microarrays in clinical settings is to construct a predictive model for diagnosis or prognosis purposes. To do so, we need to overcome the various types of noises mentioned above, especially batch effects [7]. Recently, a prominent study on how batch effect removal techniques could improve microarray prediction performance was published [8]. However, the results were not very encouraging, as the techniques studied did not always improve prediction. In fact, in up to 20% of the cases, prediction accuracy was reduced. Furthermore, it was stated in the paper that the techniques studied required sufficiently large sample sizes in both batches (train and test) to be effective, which is not a realistic situation in clinical settings.

Most batch effects removal algorithms try to accurately estimate the batch effects before removing them, which is why large sample sizes are required for each batch and a balanced class ratio is often desired. In this paper, we attack the problem from a different angle. Specifically, we propose a computational approach that increases cross-batch microarray prediction accuracy that mitigates batch effects without explicitly estimating and removing them. Our proposed approach uses the following two main ideas. Firstly, it is well known that while batch effects affect the absolute values of the gene expression measured, they often do not affect the relative ranking of the gene ordered by their expression values [5]. Thus, instead of attempting to estimate noise due to batch effects, we embrace it by using rank values rather than absolute values. Secondly, assuming the number of seriously noisy samples is far fewer than the number of relatively clean samples, we show that stochastic sampling with replacement can generate many new diverse training sets that are enriched with clean samples. Thus, instead of identifying and removing noise explicitly, we employ stochastic sampling with replacement to generate many diverse training sets that are enriched with clean samples, to suppress unwanted noise while allowing diversification to emerge.

## Materials and methods

### Data sets

**Figure 1:**
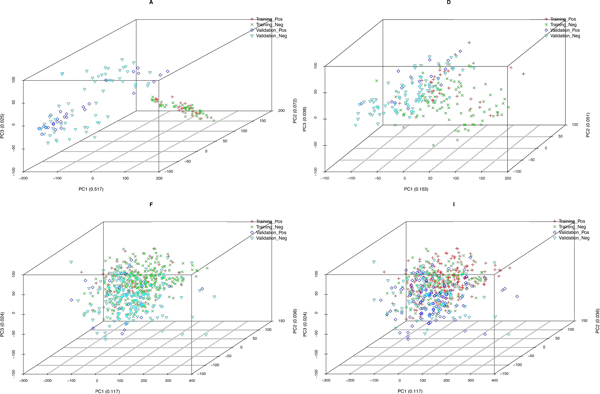
**PCA plots of data sets used**. PCA plots are typically used to visualize batch effects. These data sets are chosen from the FDA-led Microarray Quality Control (MAQC) Consortium project. See [9] for details on data sets. Based on the PCA plots, data set A contains the most batch effects (points are separated by batches instead of class labels) while data set F contains the least. Note that data set I is a negative control where class labels are randomly assigned.

Four data sets from the MAQC project are used in this paper (Table 1). Three are chosen due to their varying amount of batch effects as visually quantified using PCA (Figure 1); and the fourth one is simply a negative control where class labels were randomly assigned. We name the data sets in the same way as in the MAQC project [9].

**Table 1:** Data sets from MAQC project used in this work.

		Training set			Validation set		
	
Data set code	Data set description	Number of samples	Positives	Negatives	Number of Samples	Positives	Negatives
A	Lung tumorigen vs. nontumorigen (Mouse)	70	26	44	88	28	60
							
D	Breast cancer pre-operative treatment response (pathologic complete response)	130	33	97	100	15	85
							
F	Multiple myeloma overall survival milestone outcome	340	51	289	214	27	187
							
I	Same as data set F but class labels are randomly assigned	340	200	140	214	122	92

The Hamner Institutes for Health Sciences (Research Triangle Park, NC, USA) provided data set A. The objective of the study was to apply gene expression data from the lungs of mice exposed to a 13-week treatment of chemicals to predict increased lung tumor incidence in the two-year rodent cancer bioassays of the National Toxicology Program. Results of this study may be used to create a more efficient and economical approach for evaluating the carcinogenic activity of chemicals. A total of 70 mice were analyzed in the first phase and used as the training set. An additional 88 mice were later collected and analyzed, and subsequently used as the validation set.

The University of Texas M. D. Anderson Cancer Center (MDACC, Houston, TX, USA) generated data set D. 230 stages I-III breast cancers gene expression samples were collected from newly diagnosed breast cancers before any therapy. Specimens were collected sequentially between 2000 and 2008 during a prospective pharmacogenomics marker discovery study. Patients received 6 months of preoperative chemotherapy followed by surgical resection of the cancer. Response to preoperative chemotherapy was categorized either as a pathological complete response (pCR), which indicates no residual invasive cancer in the breast or lymph nodes, or residual invasive cancer (RD). Gene expression profiling was performed in multiple batches using Affymetrix U133A microarrays. The first 130 collected samples were assigned as the training set, whereas the next 100 samples were used as the validation set.

The Myeloma Institute for Research and Therapy at the University of Arkansas for Medical Sciences (UAMS, Little Rock, AR, USA) contributed data sets F and I. Highly purified bone marrow plasma cells were collected from patients with newly diagnosed multiple myeloma followed by gene expression profiling of these cells. The training set consisted of 340 cases enrolled on total therapy 2 (TT2) and the validation set comprised 214 patients enrolled in total therapy 3 (TT3). Dichotomized overall survival (OS) and event-free survival (EFS) were determined based on a two-year milestone cutoff.

As all the data sets above from the MAQC project are cancer-related, we have therefore gathered an additional non-cancer-related data set from a different source [10] to show that our methodology is not limited only to cancer-related data sets. This data set is a Duchenne Muscular Dystrophy (DMD) data set that compares patients suffering from DMD to normal patients. Not only does this DMD data set contains batch effects, it is also a cross-platform data set. The training set with 12 DMD patients and 12 controls comes from Affymetrix HG-U95Av2 GeneChip [11] whereas the validation set with 22 DMD pateints and 14 controls uses HG-U133A GeneChip [12]. Due to the cross-platform nature of this data set, additional pre-processing is required. Firstly, probe IDs of both chips needs to be converted into Entrez IDs and only Entrez IDs that appear on both chips are retained. Furthermore, as multiple probes could be mapped into a single Entrez ID, the maximum value of the probes are chosen to be the representative value for the Entrez ID, and this approach of collapsing is also recommended by GSEA [13].

### Proposed algorithm

As previously mentioned, we are proposing an entirely different approach towards overcoming batch effects. This computational approach is inspired by two articles in the field of biology, and is further enhanced with idea from our previous work on sequential hypothesis testing [14].

**Figure 2:**
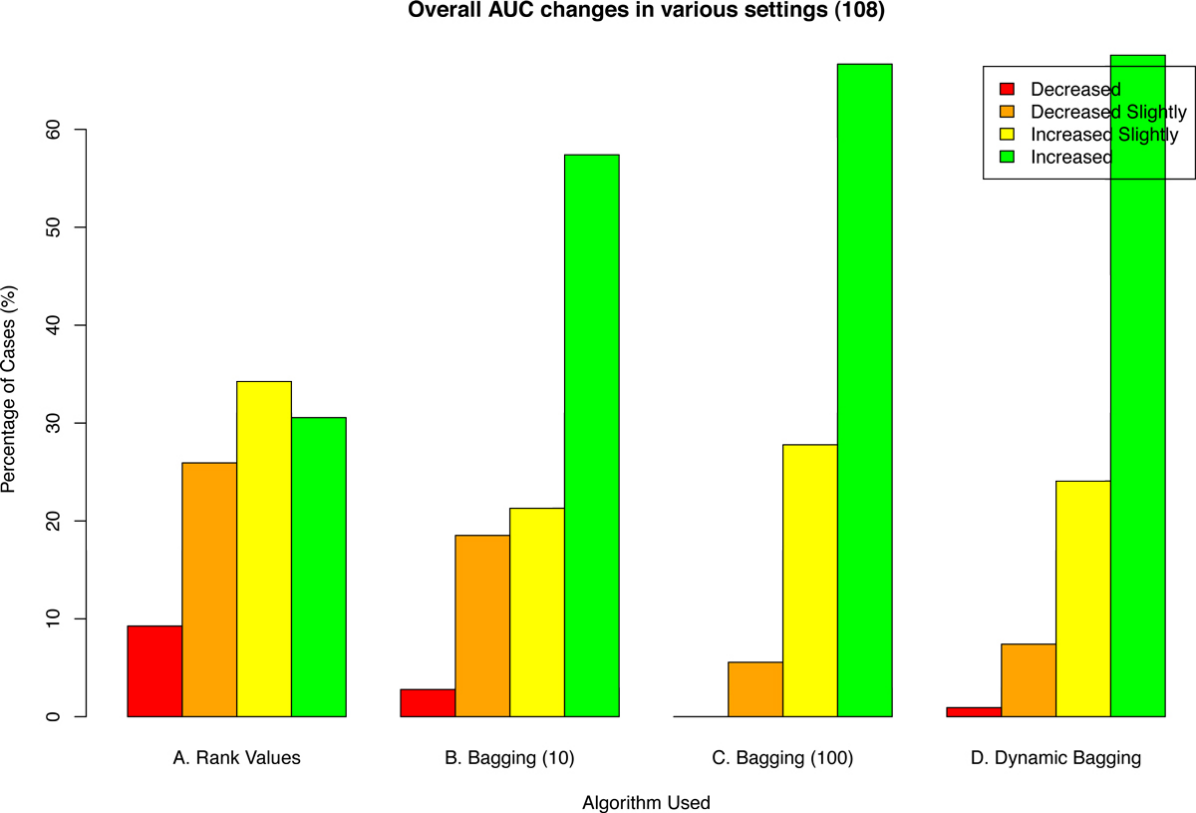
**Percentage of cases of AUC changes under various settings**. The number of scenarios explored in each setting is 108. "A. Rank Values" is using rank values instead of absolute values of microarray data. "B. Bagging (10)" and "C. Bagging (100)" are using bagging of 10 and 100 bootstrap replicates respectively with rank values. "D. Dynamic Bagging" is using bagging with non-fixed number of bootstrap replicates where the number of bootstrap replicates is determined by the sequential hypothesis testing algorithm proposed in [14] and error rates set to be 10^-4^. AUC Change = AUCafter - AUCbefore. The base AUC (i.e., AUCbefore) is where absolute gene expression values and no bagging are used. "Increased" and "Decreased" refers to cases where the change of AUC is *>*0.05 and *<*-0.05 respectively before (using absolute values) and after (using given algorithm). "Increased Slightly" is when AUC change ≥0 but ≤0.05 whereas "Decreased Slightly" indicates that AUC change *<*0 but ≥-0.05.

First, we propose using rank values instead of absolute values of gene expression microarray data. This is inspired by the FDA-led Microarray Quality Control (MAQC) Consortium project [5], where one of its findings is that while noise is inevitable in microarray experiments, the rank correlation between different experimental groups and microarray platforms remains high. It was found that gene expression data had a median coefficient of variation between 5-15% for sample replicates. In contrast, the ranks correlations (Spearman) of log ratios were highly correlated (minimum R = 0.69) even across different platforms. Therefore, by using rank values, we were able to withstand a considerable amount of experimental noise and showed improvement in performance (Figure 2).

In another article [15], a team of biologists successfully used repeated stochastic sampling to suppress experimental noise while allowing meaningful heterogeneity in a cell population to emerge. Interestingly, this approach is very similar to the bootstrapping approach in computer science. Therefore, our second idea in this algorithm is to use bootstrapping to generate numerous diverse sets of training clones from the original training data. We argue below that these training clones are likely to be enriched with more clean samples than the original training data.

**Figure 3:**
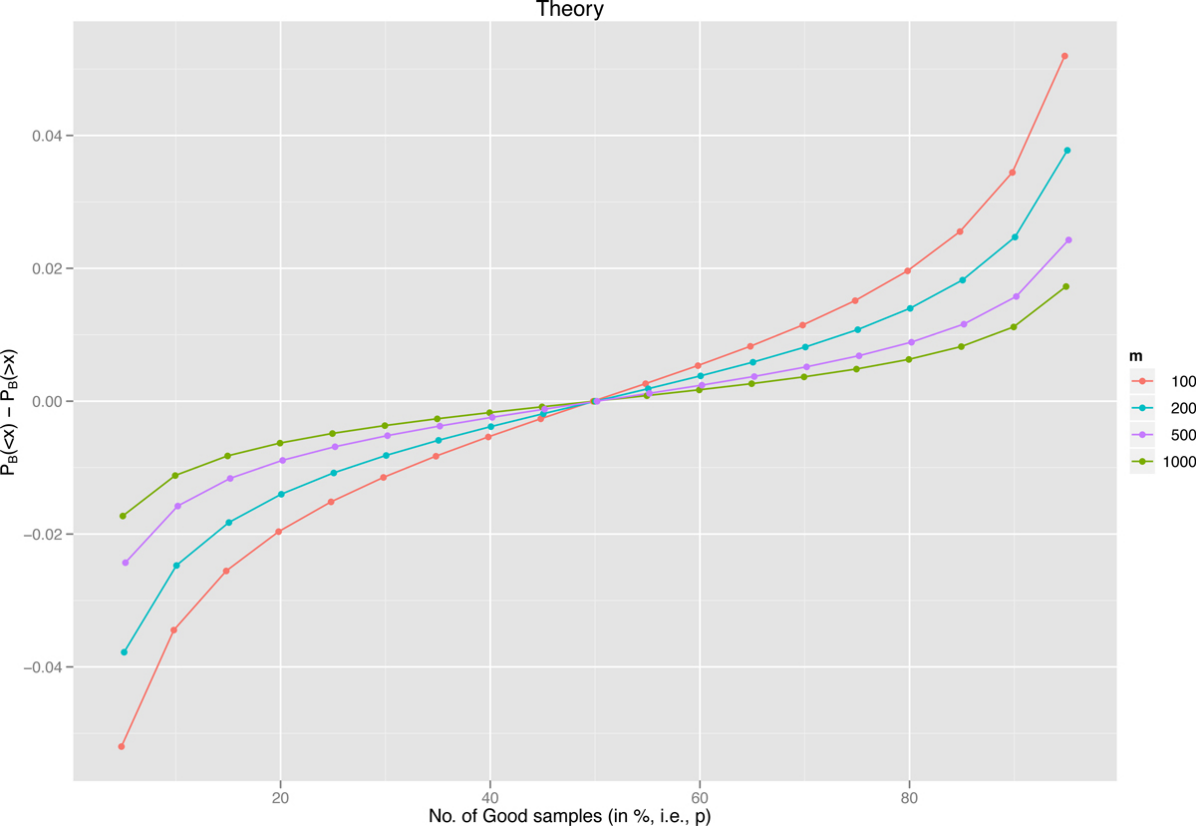
**Theoretical values of *P*_B_(*< x*) - *P*_B_(*> x*)**. Theoretical values of *P*_B_(*< x*) - *P*_B_(*> x*) for different sample size (i.e., *m*) at varying percentage of "good" samples (i.e, *p*).

Suppose a set *S *of *m *samples is given. Suppose *x *of the samples are "bad" (i.e., incorrect or very noisy) and *y *= (*m *- *x*) of the samples are "good" (i.e., correct or little noise). Let *q *= *x/m *and *p *= (1 - *q*) = (*m *- *x*)*/m*. Let *B *be a bag of *m *samples randomly drawn with repetitions from *S*. The probability of *B *having *k *"bad" samples is given by the binomial distribution *P*_B_(*k*) = (*^m^C*_k_)(*p^m-k^*)(*q^k^*), where *^m^C_k _*means "*m *choose *k*". Then the probability of *B *having fewer "bad" samples than *S *is given by $P_{B}\left(<x\right)=\sum_{k<x}P_{B}\left(k\right)$, while the probability of *B *having more "bad" samples than *S *is given by $P_{B}\left(>x\right)=\sum_{k>x}P_{B}\left(k\right)$. The skewness of a binomial distribution is given by the formula $\left(1-2q\right)/\sqrt{mqp}$. When *p *>*q*, and thus 1 > 2*q*, the skew is positive. This means that, in general, the bulk of the distribution falls to the left of the mean *x *= *mq*. Thus, *P*_B_(*<x*) *> P*_B_(*>x*), as shown in Figure 3.

Therefore, we have shown that training clones produced using bootstrapping technique are likely to be enriched with more clean samples than the original training data.

We further show that an ensemble classifier built using these training clones has better performance. Let *B*_1_, *B*_2_, *..*.,*B_n _*be *n *bags of *m *samples randomly drawn with repetitions from *S*. Let *H *be the collection of bags among *B*_1_, *B*_2_, *..*.,*B_n _*containing more "good" samples than *S*. Let C(*B*_1_) and C(*B*_2_) be the classifiers trained on *B*_1 _and *B*_2 _respectively. It is reasonable to postulate (*) that C(*B*_1_) would have a better accuracy than C(*B*_2_) if *B*_1 _has more "good" samples than *B*_2_. Let *H' *be the collection of bags among *B*_1_,*B*_2_, *..*.,*B_n _*containing more "bad" samples than *S*. Let *H" *be the collection of bags among *B*_1_,*B*_2_, *..*.,*B_n _*containing the same number of "good" and "bad" samples as *S*. Based on the postulate (*), *h *= |*H*| bags give rise to better-performing classifiers than *C*(*S*), while *h' *= |*H' *| bags give rise to poorer-performing classifiers, and the remaining *h" *= |*H" *| bags give rise to equal-performing classifiers. We know that as *n *tends to infinity, *h/n *tends to *P*_B_(*<x*), *h'/n *tends to *P*_B_(*>x*), and *h"/n *tends to *P*_B_(*x*). It follows that *h *>*h'*, when *p *>*q*.

This shows that an ensemble classifier built from this collection of bags--which is called a bagging classifier--improves prediction accuracy by embracing (i.e., reducing the influence of) noisy samples, as long as there are many more "good" samples than "bad" samples in the original training set *S*, which is a reasonable assumption for any decent training datasets.

However, in the original flavor of bagging, while *n *needs to be sufficiently large to ensure *h *≥ *h'*, *n *is typically determined a priori and arbitrarily [16]. Here, we propose integrating bagging with a sequential hypothesis testing procedure [17], which then allows us to dynamically determine the optimal *n *required for each test instance. In our previous work [14], we developed a sequential hypothesis testing procedure called OSM (Optimized Statistical Model Checking Algorithm). OSM is able to determine the number of simulation runs required to prove whether a stochastic model satisfies a probabilistic formula, *P*_≥*θ*_{*ψ*} where *θ *represents the threshold probability and *ψ *represents the property. For instance, *P*_≥0.8_{*X*_1_*>*5} checks whether the given stochastic model would have variable *X*_1 _*>*5 in ≥ 80% of the cases. Essentially, this procedure draws samples until it can assert or reject a probabilistic formula with statistical guarantees on the error rates.

Let *T_i _*be the *i*^th ^test instance and *P*(*C*(*B_n_*), *T_i_*) be the Boolean prediction on *T_i _*of the classifier trained using *B_n_*, which will return *True *if *T_i _*is predicted to be of the positive class label and *False *if *T_i _*is predicted to be of the negative class label. We can then formulate the probabilistic formula as *P*_≥0.5_{*P*(*C*(*B_n_*), *T_i_*) = *True*}. That is, given a test instance *T_i _*and a training set *S*, and a large number of classifiers trained from bags of *m *samples randomly drawn with repetitions from *S*. Would more than 50% of these classifiers predict *T_i _*to be of the positive class label? By stating the problem in this format, *n *is dynamically determined by the sequential hypothesis testing procedure and is minimum for each test instance with statistical guarantees on the error rates. This improves computational efficiency and maintains prediction accuracy over standard bagging. Furthermore, this removes the need to a priori and arbitrarily fixing *n*. For the purpose of comparison with standard bagging (with 10 and 100 bootstrap replicates), we set the parameters for the OSM sequential hypothesis algorithm as follows. The maximum value of *n *is set to 100 and the guaranteed false positive and false negative error rates are both set to 10^-4^.

In summary, we propose using ranking value of microarray data and bagging with sequential hypothesis testing to dynamically determine the number of classifiers required. Finally, the average of these classifiers scores is taken as the final prediction score for a particular test instance.

### Evaluation of effectiveness

In this work, our main objective is to improve cross-batch prediction accuracy. Therefore, we will be using it as our performance measurement. The primary performance metric used will be area under the ROC curve (AUC) as it has the advantage of evaluating performance across the full range of sensitivity and specificity. A prediction model will be built using the training set and evaluated using the validation set (forward prediction) and vice versa (backward prediction).

To demonstrate the applicability of our proposed algorithm in small-sample-size scenarios, we create two additional data sets by randomly selecting 25% or 50% of the samples while maintaining the class ratio from each of the original data sets given in Table 1. In total, we have 12 training sets and 12 validation sets. Next, in order to show that our approach is independent of the feature selection algorithm and classification methods, we have chosen several different approaches representing various categories. For feature selection, we have picked *t*-test and Wilcoxon Rank Sum test as they represent parametric and nonparametric approaches respectively. As for classification methods, we have chosen support vector machine (SMO with buildLogisticsModel set to True), K nearest neighbors (*K *= 5) and the popular tree algorithm, *C*4.5 (named as J48 in Weka [18]). All classification methods uses the default settings in Weka 3.6.4 with the stated changes. They represent linear classifier, instance-based classifier and tree classifier respectively.

Using the above-mentioned data sets, feature selection algorithms and classification methods, we measure the difference in AUC before and after our proposed algorithms in each of the possible permutations. There are a total of 9 different data sets, 3 from each data set A, D and F. Data set I is not used to measure performance improvement since it is a negative control; it is used instead to ensure arbitrary improvement is not seen. Together with two different prediction directions (forward and backward), two different feature selection algorithm (t-test and Wilcoxon Rank Sum test) and three different classification methods (SVM, k-NN and C4.5), there are a total of 108 (9x2x2x3) different possible scenarios.

## Results

**Figure 4:**
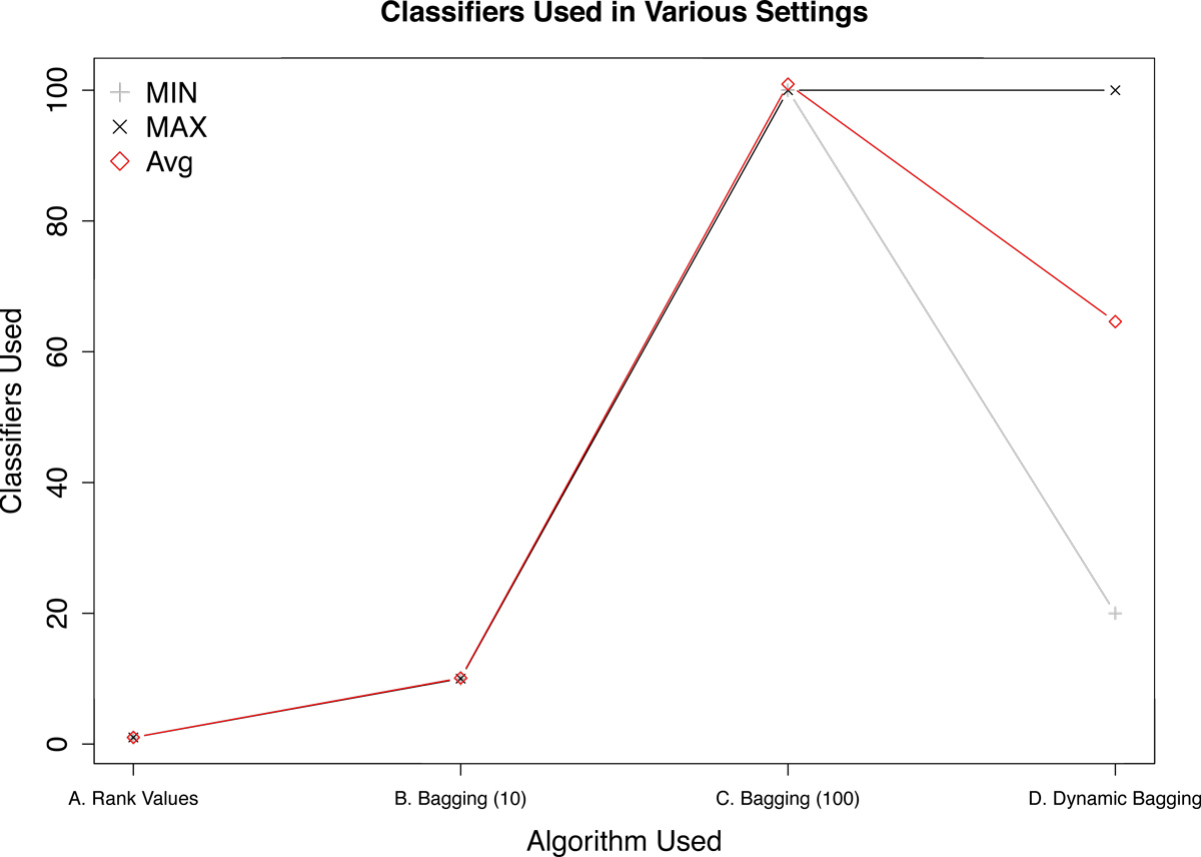
**Classifiers used by various settings**. "A. Rank Values" is using rank values instead of absolute values of microarray data. "B. Bagging (10)" and "C. Bagging (100)" are using bagging of 10 and 100 bootstrap replicates respectively with rank values. "D. Dynamic Bagging" is using bagging with non-fixed number of bootstrap replicates where the number of bootstrap replicates is determined by the sequential hypothesis testing algorithm proposed in [14] and error rates set to be 10^-4^. "MIN" is the minimum number of classifiers used in all scenarios. "MAX" is the maximum number of classifiers used in all scenarios. "AVG" is the average number of classifiers used in all scenarios. The number of scenarios explored in each setting is 108.

The main objective of this work is to improve cross-batch prediction performance. In Figure 2, we looked at the AUC change in all 108 possible permutations for various algorithms. Figure 2 shows that our proposed algorithm is able to improve AUC by *>*0.05 in *>*60% of the cases with only one case (*<*2%) having reduced AUC of exceeding -0.05. Combining the observations of Figure 2 and 4, one can easily infer that having more classifiers in the ensemble for majority voting would increase performance, but having more classifiers would also require additional computational resources. The number of bootstrap replicates to use is typically decided arbitrarily. This is where dynamic bagging has an edge; it would use just enough classifiers to make the prediction. In Figures 2 and 4, it is demonstrated that dynamic bagging is able to achieve a comparable performance using only about 60% of the number of bootstrap replicates on average as compared to bagging with 100 bootstrap replicates.

**Figure 5:**
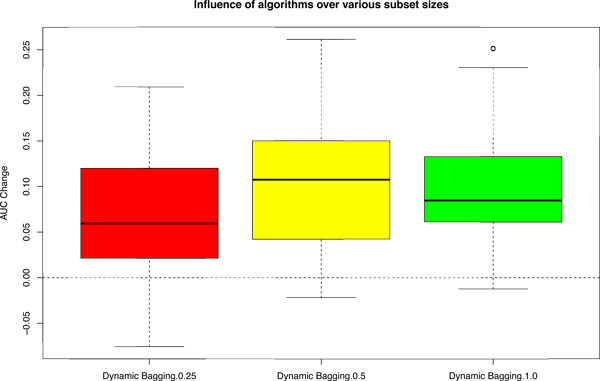
**Boxplot of AUC change on varying subset sizes under various scenarios (36) for data set A, D and F**. AUC Change = AUCafter - AUCbefore. Subset size here implies using a random subset of the given data during training phase. "Dynamic Bagging.0.25", "Dynamic Bagging.0.5" and "Dynamic Bagging.1.0" are the AUC change after applying dynamic bagging and using rank values with 25%, 50% and 100% of the original given data for training respectively compared with the conventional approach, which is without bagging and using absolute values [9].

Another important consideration in building prediction models for clinical usage is the required sample size of training and test sets to properly deploy it. As the MAQC project is a large-scale study, its data sets are larger than usual. We did random subset sampling to reduce the number of samples available to us to as low as 25% of the original data, during the training phase, to mimic the low sample size in clinical settings. Despite the reduction in training samples, our algorithm still maintained its improvements with median AUC improvements well above 0.05 (Figure 5). It is worth noting that the number of samples in the test data set has no influence on prediction performance for our algorithm since we use them individually and solely for the purpose of classifying it unlike conventional batch removal methods.

**Figure 6:**
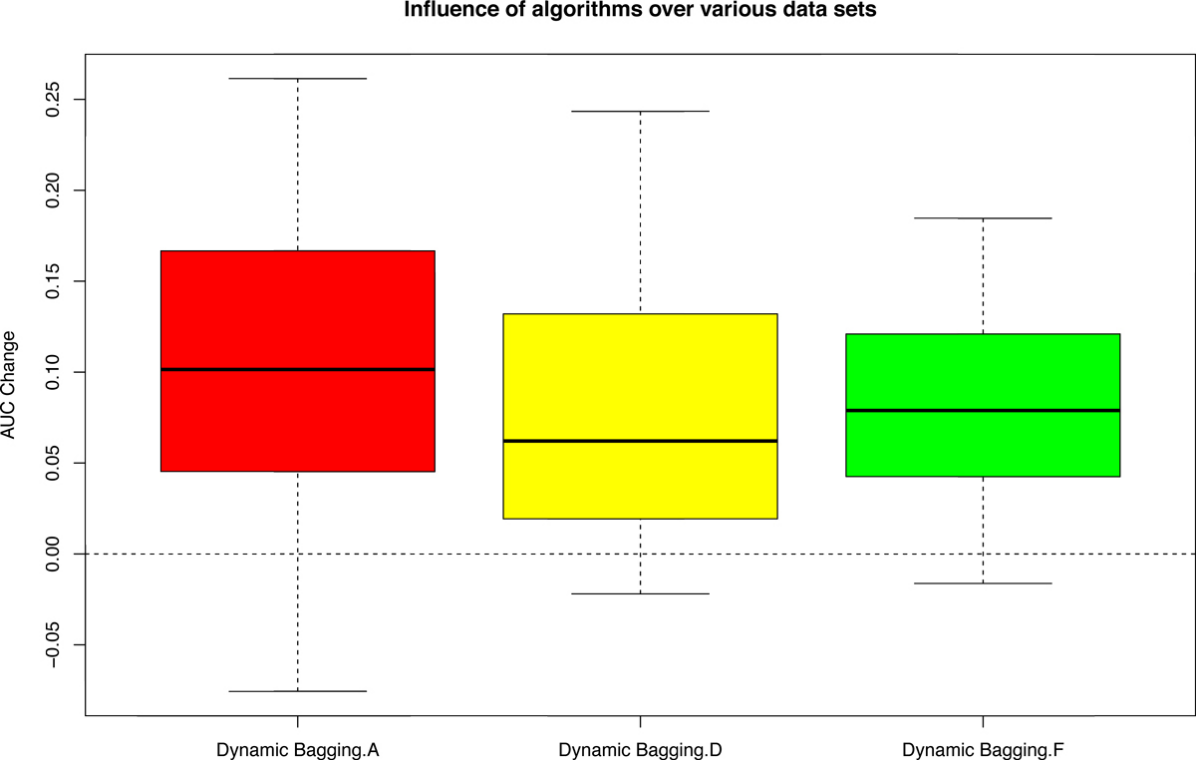
**Boxplot of AUC change on different data sets (A, D, F) under various scenarios (36)**. AUC Change = AUCafter - AUCbefore. "Dynamic Bagging.A", "Dynamic Bagging.D" and "Dynamic Bagging.F" are the AUC change after applying dynamic bagging and using rank values on data sets A, D and F respectively compared with the conventional approach, which is without bagging and using absolute values [9].

As the PCA plots of Figure 1 suggested that the different data sets are likely to have a varying amount of batch effects, it is also interesting to look at how our algorithm would perform on each data set. Figure 6 shows that our proposed algorithm performs consistently with median AUC improvement well above 0.05 regardless of the data set. This consistent improvement across various data sets with varying "magnitudes" of batch effects implies that our algorithm is able to successfully overcome batch effects.

**Figure 7:**
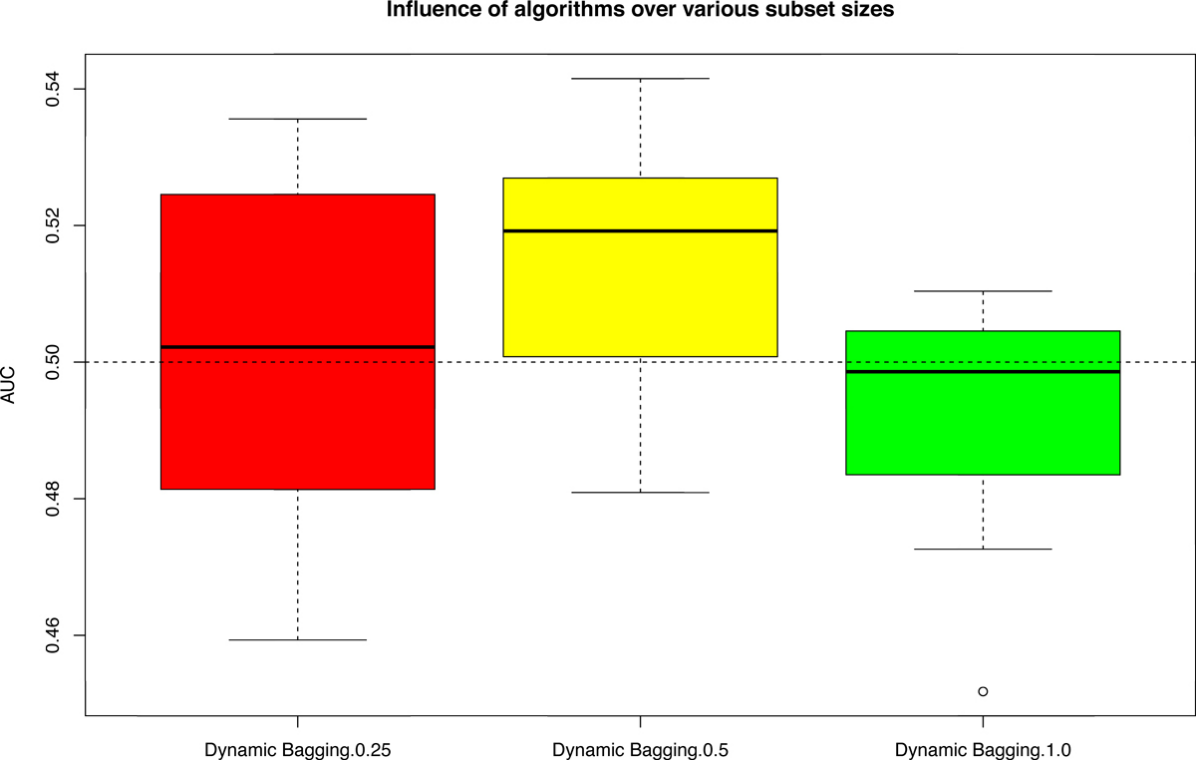
**Boxplot of AUC on varying subset sizes under various scenarios (36) for data set I**. Subset size here implies using a random subset of the given data during training phase. "Dynamic Bagging.0.25", "Dynamic Bagging.0.5" and "Dynamic Bagging.1.0" are the AUC achieved by applying dynamic bagging and using rank values with 25%, 50% and 100% of original given data for training.

Finally, one critical issue highlighted by the MAQC project [9] is regarding proper validation procedure to ensure the independence of the validation set, such as modification of an originally designed algorithm after being validated on the validation set. This would turn the validation set into part of the training process. To ensure that our algorithm is not arbitrarily improving performance, we test it on a negative control data set (data set I). Since it is a negative control data set, the AUC should be close to 0.5 and, as shown by Figure 7, after applying our algorithm, the median AUC is very close to 0.5 and the distribution is within a tight range of 0.45 to 0.55. This conclusively shows that our algorithm does not arbitrarily inflate performance.

## Additional validation

**Figure 8:**
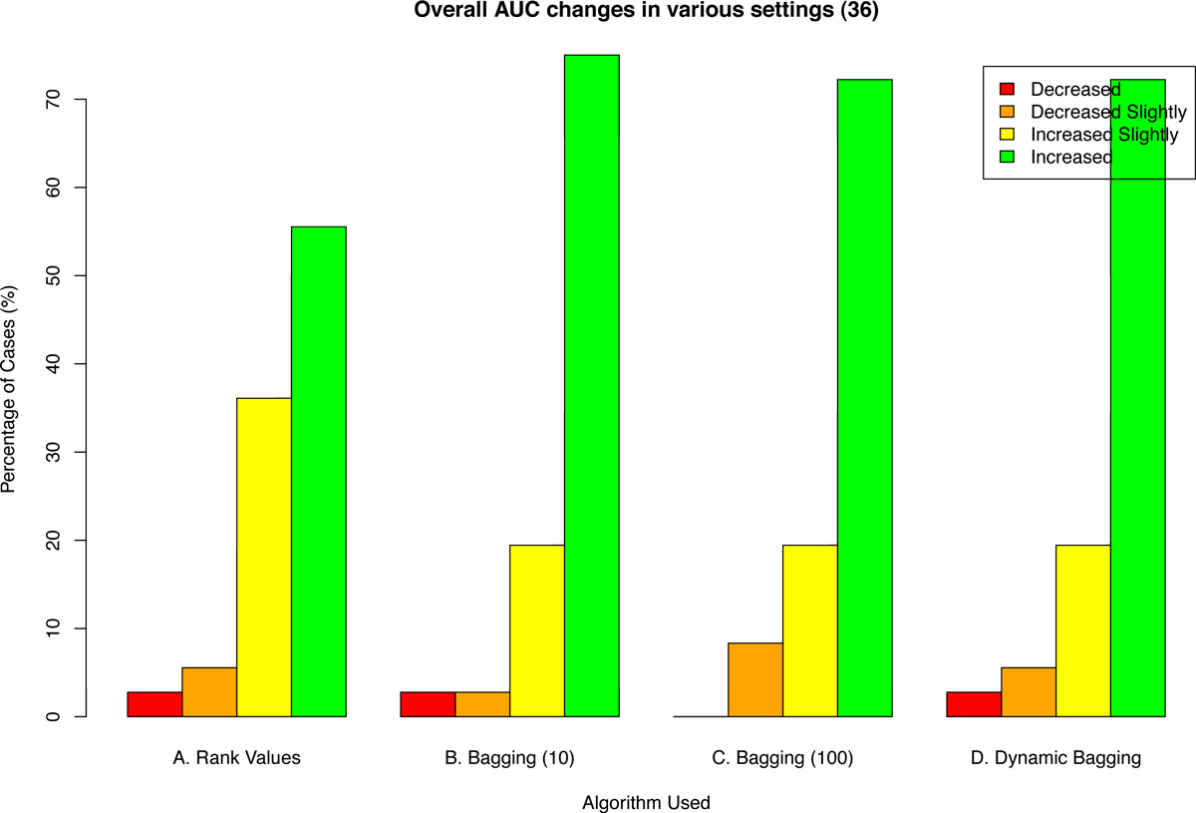
**Percentage of cases of AUC changes under various settings for DMD data set**. The number of scenarios explored in each setting is 36. "A. Rank Values" is using rank values instead of absolute values of microarray data. "B. Bagging (10)" and "C. Bagging (100)" are using bagging of 10 and 100 bootstrap replicates respectively with rank values. "D. Dynamic Bagging" is using bagging with non-fixed number of bootstrap replicates where the number of bootstrap replicates is determined by the sequential hypothesis testing algorithm proposed in [12] and error rates set to be 10^-4^. AUC Change = AUCafter - AUCbefore. The base AUC (i.e., AUCbefore) is where absolute gene expression values and no bagging are used. "Increased" and "Decreased" refers to cases where the change of AUC is *>*0.05 and *<*-0.05 respectively before (using absolute values) and after (using given algorithm). "Increased Slightly" is when AUC change ≥0 but ≤0.05 whereas "Decreased Slightly" indicates that AUC change *<*0 but ≥-0.05.

In addition to cancer-related data sets from MAQC projects, we have also obtained a DMD data set from a different source [10] to demonstrate that our methodology is not limited to a specific group of problems. The conclusion that we obtained from running our methodology on the DMD data set is similar to the MAQC data sets (Figure 8). By simply using ranking values instead of absolute values, significant improvements can be seen. Complimenting that with bagging brings the improvements one notch higher, while dynamic bagging is able to maintain high performance with a minimum number of bootstrap replicates. With this DMD data set, we have shown that our methodology works well also on a non-cancer-related data set and it further suggests that our work is able to overcome cross-platform prediction problems in addition to batch effects.

## Discussion

Overcoming batch effect is an important step before the deployment of diagnostic or prognostic model based on gene expression data in clinical settings. Numerous algorithms have been proposed in an attempt to solve this widespread and critical problem in high-throughput experiments [19-21]. However, these algorithms typically focused on accurately estimating batch effects and then removing them using various methodologies. Often, the applicability and efficacy of these algorithms rely heavily on the sample size and class ratio of each individual batch. This prevents the methods from being applicable in clinical settings, where batch size is likely to consist of only a few single samples. While the use of calibration samples might somehow be able to overcome this, we also need to consider the other pertinent issues such as additional costs and proper preservation procedures.

In this work, we approached the batch effects problem from a different angle. We proposed a computational algorithm that attempts to embrace noise instead of estimating and removing it. By simply employing the ranking of values instead of using the absolute values of data, we were already able to show noticeable improvements. Combining this with bagging and a sequential hypothesis-testing algorithm; we were able to achieve a significant increase in cross-batch prediction performance over a wide range of training data sample size and severity of batch effects. It is important to note that our approach does not face the same limitations as conventional batch effects removal methods; thus making it appealing for use in practical applications.

Feature selection algorithms considered in this work use only generic statistical tests that look at one gene at a time. However, more recent feature selection algorithms for gene expression data are increasingly focused on using prior biological information to group genes and perform statistical tests on these group of genes instead of individual genes [10,13]. The impact of such algorithms is not evaluated in this work and would be considered for future work. Another interesting possible future work would be to explore the impact of dynamic bagging in other fields. Bagging is a widely used ensemble algorithm to improve classification accuracy. However, deciding the number of bootstrap replicates is typically done a priori and arbitrarily. It would be interesting to study whether our dynamic bagging technique would be equally successfully when applied to other fields, which we believe to be highly possible, as we did not incorporate any biology-specific assumption in its derivation.

## Competing interests

The authors declare that they have no competing interests.

## Authors' contributions

Both authors contributed equally to conceiving the proposed method. C.H.K carried out the programming, prepared and ran the experiments, and drafted the manuscript. L.W designed the experiments and played a supervision role for the study. Both authors read and approved the final manuscript.
